# Informing the American Academy of family Physician’s Health Equity strategy – an environmental scan using the Delphi technique

**DOI:** 10.1186/s12939-019-1007-1

**Published:** 2019-06-21

**Authors:** Kevin A. Kovach, Cory B. Lutgen, Elisabeth F. Callen, Christina M. Hester

**Affiliations:** 1 American Academy of Family Physicians, 11400 Tomahawk Creek Parkway, Leawood, KS 66211 USA; 2 National Research Network, American Academy of Family Physicians, 11400 Tomahawk Creek Parkway, Leawood, KS 66211 USA

**Keywords:** Health Equity, Social determinants of Health, Expert panel, Delphi technique, Medical specialty society, Strategic planning

## Abstract

**Background:**

Many organizations have prioritized health equity and the social determinants of health (SDoH). These organizations need information to inform their planning, but, relatively few quantifiable measures exist. This study was conducted as an environmental scan to inform the American Academy of Family Physician’s (AAFP’s) health equity strategy. The objectives of the study were to identify and prioritize a comprehensive list of strategies in four focus areas: health equity leadership, policy, research, and diversity.

**Methods:**

A Delphi study was used to identify and prioritize the most important strategies for reducing health inequities among the four aforementioned focus areas. Health equity experts were purposefully sampled. Data were collected in three rounds for each focus area separately. A comprehensive list of strategy statements was identified for each focus area in round one. The strategy statements were prioritized in round two and reprioritized in a final third round. Quantitative and qualitative data were integrated for the final analysis.

**Results:**

Fifty strategies were identified across the four focus areas. Commitment to health equity, knowledge of health inequities, and knowledge of effective strategies to address the drivers of health inequities were ranked the highest for leadership. Universal access to health care and health in all policies were ranked highest for policy. Multi-level interventions, the effect of policy, governance, and politics, and translating and disseminating health equity interventions into practice were ranked the highest for research. Providing financial support to students from minority or low-socioeconomic backgrounds, commitment from undergraduate and medical school leadership for educational equity, providing opportunities for students from minority or low-socioeconomic backgrounds to prepare for standardized tests, and equitable primary and secondary school funding were ranked highest for diversity.

**Conclusions:**

The AAFP and other medical specialty societies have an important opportunity to advance health equity. They should develop a health equity policy agenda, equip physicians and other stakeholders, use their connections with practice-based research networks to identify and translate practical solutions to address the SDoH, and advocate for a more diverse medical workforce.

**Trial registration:**

Not applicable.

**Electronic supplementary material:**

The online version of this article (10.1186/s12939-019-1007-1) contains supplementary material, which is available to authorized users.

## Background

Health inequities are defined as differences in health outcomes that are systematic, avoidable, and unjust [1]. Health inequities are largely created by social factors, such as governmental decision-making, public policy, culture, racism, as well as differences in power, class, and access to resources – commonly referred to as the social determinants of health (SDoH) [2]. Social factors have been referred to as “risk regulators” or the “causes of the causes” of poor health and have led to excessive negative impacts on population health and substantial health inequities by placing constraints on people’s opportunities to be healthy and affecting people’s regulatory systems through chronic stress and allostatic load [3–5]. In recent years, the impact of the SDoH in the United States has become more apparent; life expectancy decreased for the first time in decades, and diseases of despair, including suicide, alcohol abuse, and drug addiction have risen dramatically since the 1990s [6, 7]. These changes are in addition to long standing differentials in black and white infant mortality, up to 20 year differences in life expectancy between counties, and other health disparities in almost all measures of health status [8–10].

The demonstrable but complex influence of SDoH on varying outcomes brings into question the direction society at large should take to ensure the highest achievable health for all people [11, 12]. Intersectoral and cross-disciplinary approaches such as health in all policies, community health assessment and improvement planning, public-private partnerships, academic-community collaboration, and various other efforts to engage disadvantaged communities directly and work towards innovative solutions built on the diverse strengths of partnering organizations have been called for and are becoming more commonplace [13–16]. Many medical and public health organizations have prioritized health equity and SDoH [17] in response to data showing that social factors have the largest impact on population health [18], yet receive comparatively little funding relative to other factors [19]. Organizations need to be well-informed about the best possible direction for their health equity strategies to maximize their impact. Currently there are many recommendations and calls to action to advance health equity. The World Health Organization’s Commission on the SDoH have made overarching recommendations to improve daily living conditions; address the inequitable distribution of power, money, and resources; measure and understand the problem; and assess the results of action [20]. The National Academy of Medicine’s and Robert Wood Johnson Foundation’s Culture of Health Program have also provided a call to action to build “a culture of health movement by empowering communities and working across sectors and disciplines [21].” And while there is a plethora of information highlighting existing health disparities and general calls to action, there are relatively few evidence-based recommendations for more specific interventions, programs and policies to improve health equity. This can make it challenging to prioritize and create a coherent strategy that aligns organization’s strengths with health equity needs.

In 2017, the American Academy of Family Physicians (AAFP) adopted a strategic priority to become a leader in addressing SDoH and advancing health equity, by establishing its Center for Diversity and Health Equity (CDHE) [22, 23]. The CDHE has goals to educate, equip and empower family physicians about the SDoH and their impact on health and health equity. The CDHE also aims to advocate for health equity; build collaborative partnerships at the national, state, and local levels; promote greater diversity in medicine; and shape the research agenda for health equity in family medicine [23]. These strategies are intended to connect the AAFP’s strengths with opportunities to address the SDoH and improve health equity. Family physicians can be important health equity champions – a crucial piece of health equity capacity [24]. AAFP is a strong advocate for public health; public policy is a major contributing factor to health inequities [25]. The medical profession is much less diverse than the nation as a whole, and physicians from non-white and lower socio-economic backgrounds are more likely to serve and provide higher quality care in these communities [26]. More research is needed to find, translate, and disseminate solutions to advance health equity [10].

The AAFP conducted this study as part of an environmental scan to clarify and shape their health equity strategy. Bryson states that an environmental scan is used to identify internal strengths and weaknesses and external opportunities and threats [27]. The primary purpose of this study was to identify and prioritize opportunities for focusing the AAFP’s health equity strategy in a way that aligned with their strengths. This was accomplished by identifying the most important strategies for reducing health inequities and addressing the SDoH as determined by health equity experts from medicine and public health. Focus areas for the study included the following research questions:

- What skills and characteristics do physicians need to become leaders for health equity?
- What public policies are the most important for improving health equity?
- What are the most important areas of research to advance health equity?
- What are the most important policies and practices to increase diversity in medicine?

The objectives of the study were to identify and prioritize a comprehensive list of strategies for each of the focus areas and provide context and rational for the experts’ decisions. While this study was done primarily to inform the AAFP, the findings may be applicable to other organizations seeking to develop plans to advance health equity.

## Methods

### Design

**Fig. 1:**
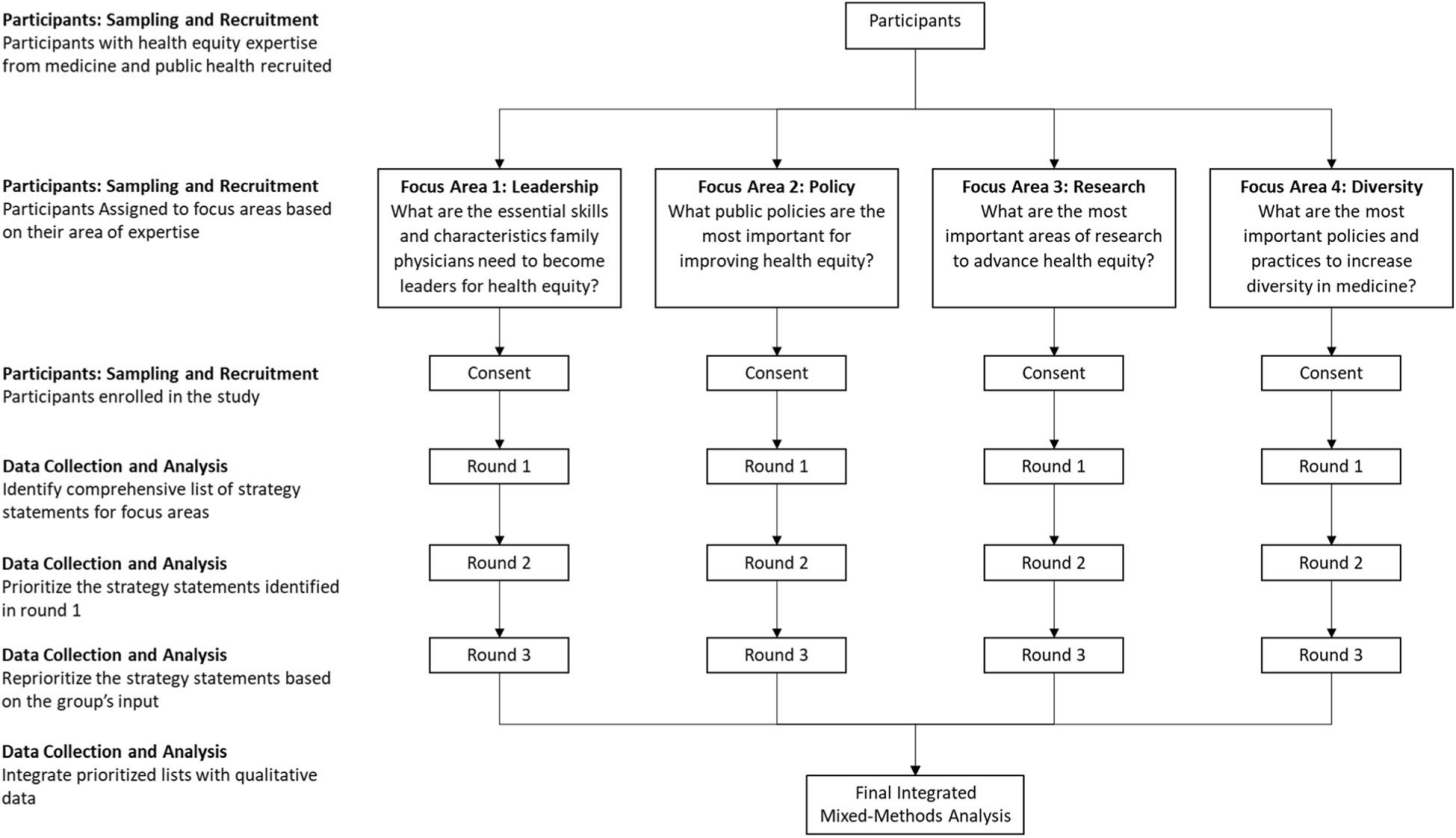
Study Design – Delphi Technique

The Delphi technique was used to identify and prioritize the most important strategies for reducing health inequities among the four aforementioned focus areas: leadership, policy, research, and diversity. The Delphi technique is a group communication process that allows experts to address complex problems without meeting face-to-face [28]. This technique has been used for a variety of purposes, including strategic planning [29] and priority setting [30, 31]. The Delphi technique is appropriate when subjective judgements from experts are useful to address complex issues that lack a clear factual basis for decision making. In addition, the technique allows experts from various disciplines and geographic regions to be convened in a way that reduces the risk of dominance by a small number of vocal participants [28]. For these reasons, the Delphi technique was deemed appropriate for this study. The overall study design is described in detail in the following sections (see Fig. 1 for study design structure). This study was reviewed by the American Academy of Family Physician’s Institutional Review Board and was deemed exempt under 45 CFR 46.101(B) - (HRP-312) Category 2.

### Participants: sampling and recruitment

Individuals were recruited to the study based on their ability to provide meaningful insight into one or more of the focus areas (leadership, policy, diversity, and research). Every recruit was employed in a position requiring substantial expertise in principles important to health equity (social justice, engaging disadvantaged populations, advocacy, promoting diversity in medicine, increasing access to care, etc.). Participants came from various types of organizations, including national medical specialty societies and public health associations, medical schools and schools of public health, the Federal government, as well as medical practice and public health service. Every participant held an advanced degree (e.g., Doctor of Medicine, Doctor of Osteopathic Medicine, Doctor of Philosophy, Juris Doctorate, Master of Public Health, or other master’s degree). In addition to these qualifications, participants were recruited for their specific knowledge related to one or more of the focus areas. The leadership focus area included family physicians and individuals with experience collaborating with the health care sector to identify the skills and characteristics family physicians need to be health equity leaders. The policy focus area included individuals involved in policy development and advocacy to identify public policies needed to advance health equity. The research focus group included social epidemiologists, primary care researchers, and individuals responsible for translating evidence into practice to identify health equity research gaps. The diversity focus area included individuals involved in primary and secondary education, as well as medical education to identify policies and practices that could increase diversity in medicine Expertise was ascertained based on a combination of academic preparation, research and publication, service and leadership, as well as job experience.

Participants were recruited by email. Each participant was sent an introductory message that described the purpose and procedures of the study, the focus areas they were asked to respond to (a maximum of two), and a consent form. Participants that completed the consent form were enrolled in the study. The goal was to enroll 10 participants per focus area for each round. This goal was chosen because previous research on the Delphi technique suggests this is a minimum threshold, as well as the relatively rapid needs of the environmental scan and the number of focus areas being explored [32]. Forty-one individuals were recruited in round one and 66 individuals were recruited in rounds two and three to bolster the number of participants.

### Data collection and analysis

SurveyMonkey was used to administer the surveys [33]. Data were collected in three rounds for each focus area separately, occurring over a three-month period in the Spring of 2018. Participants were asked to complete the survey in 2 weeks; however, up to 6 weeks were provided to increase the response rate. Three reminder emails were sent per round to increase the response rate. Individuals that completed a round were invited into the next round. Additional individuals were recruited if fewer than 10 individuals participated in a round.

#### Round one

The purpose of round one was to identify a comprehensive list of strategy statements related to each focus area that would be used in rounds two and three. To accomplish this, participants were asked an open-ended question about which strategies they thought were important to the focus area and to explain why they thought these strategies were important. The specific questions asked in each focus area were:

- Leadership: What are the essential skills and characteristics family physicians need to become leaders for health equity?
- Policy: What public policies are the most important for improving health equity?
- Research: What are the most important areas of research needed to advance health equity?
- Diversity: What are the most important policies and practices to increase diversity in medicine?

Participants were provided a definition of health equity and health inequities. For the study, health equity was defined as the “highest attainment of health for all people,” and health inequity was defined as the “differences in health that are avoidable, unfair, and unjust, and that are driven by social, economic, and environmental conditions.” These definitions were informed by the American Public Health Association and The Centers for Disease Control and Prevention [1, 34].

The participants’ input was used to develop a list of strategy statements to be used in round two. When participants provided clear and complete thoughts about a strategy, the language was used with only minor edits to develop the strategy statement. When participants provided incomplete or truncated thoughts about a strategy, the language for the strategy statement was informed by a literature review of systematic reviews, research articles, and editorials [12, 35–39]. Eighty-six strategy statements were provided in total (leadership: 17; policy: 34; diversity: 18; and research: 17) (Additional file 1).

#### Round two

The primary purpose of round two was to begin to prioritize the strategy statements identified in round one. Strategy statements were presented in random order to reduce order effect bias. Participants were asked to rate each strategy statement on a seven-point ordinal scale (1 = Not a Priority, 2 = Low Priority, 3 = Medium-Low Priority, 4 = Medium Priority, 5 = Medium-High Priority, 6 = High Priority, 7 = Essential). The research team suspected that participants may have high approval for every strategy statement and would rank them as all being priorities. This was in fact the case, and all but two of the 86 strategy statements were rated as being a priority (mean rating ≥ 4). To address the lack of variance, we also asked participants to rank the top three most important strategy statements. Bradburn and colleagues suggest using this type of approach when approval is expected to be high and when the list of statements is large [40]. The research team calculated mean and median ratings for all the strategy statements. A rank score was also calculated for all the strategy statements by assigning points to the rankings (three points for first, two points for second, and one point for third) and taking the sum across participants. Strategy statements that were ranked in the top three by any participant were retained for round three.

#### Round three

The purpose of round three was to finalize the prioritization of the strategy statements. Strategy statements were presented in order of the rank score from most to least important to reinforce the group’s input from round two. Ordinal scales were not used in this round due to the high approval observed in round two. Participants were asked to rank each strategy statement from most to least important in accordance with recommendations from Bradburn and colleagues [40]. Participants were also asked why they thought the statements were important, and what the most important things the AAFP should do for each focus area.

### Final analysis

The data were analyzed by integrating quantitative rankings with qualitative data. For each focus area, the average rank and standard deviation for each statement were calculated using results from round three. Statements were ordered from the most to least important. Priority among ties was given to statements with a smaller standard deviation. The interquartile interval was calculated for the average rank to further stratify the statements.

Qualitative data from each round of the study were used to provide context and meaning to the prioritized lists [41, 42]. The research team used a deliberative and iterative process for the final analysis. Quotations were coded to inventory their meaning. Two researchers (KK and CL) worked together to clarify the meaning of the codes and come to agreement on codes. The entire research team further deliberated until consensus was reached. Quotations were then integrated with the prioritized lists by identifying statements raised in the qualitative data, selecting representative quotations, and triangulating this with the prioritized lists.

## Results

### Participant characteristics

Twenty-seven individuals participated in this study (response rate, 41%), with 23 (response rate, 56%), 25 (response rate, 38%), and 24 (response rate, 36%) participating in rounds one, two, and three, respectively. On average, experts participated in 2.7 rounds. Sixteen (59%) participants were female and eleven (41%) were male. Eight (30%) participants held a Doctor of Medicine/Osteopathic Medicine plus another master’s degree or doctorate, seven (26%) held a Doctor of Philosophy, six (22%) held a Doctor of Medicine/Osteopathic Medicine, four (15%) held a master’s degree, and two (7%) held a Juris Doctorate. Sixteen (59%) participants worked for a university, five (19%) worked for an association, five (19%) worked in medical or public health practice, and one (4%) worked for the Federal Government. Among the participants: eleven (41%) participated in the leadership panel; ten (37%) participated in the policy panel; eight (30%) participated in the research panel; and nine (33%) participated in the diversity panel. Sixteen participants participated in one focus area while eleven participated in two. This information is presented in Table 1.

**Table 1:** Participant Characteristics

	Round 1		Round 2		Round 3		All Rounds	
Panel Characteristics	N	%	N	%	N	%	N	%
Gender								
Female	13	57	15	60	15	63	16	59
Male	10	43	10	40	9	38	11	41
Education								
MD/DO & Master’s or Doctorate	7	30	7	28	6	25	8	30
PhD	7	30	6	24	6	25	7	26
MD/DO	5	22	6	24	6	25	6	22
Master’s Degree	2	9	4	16	4	17	4	15
JD	2	9	2	8	2	8	2	7
Institutional Affiliation								
University	15	65	14	56	14	58	16	59
Association	2	9	5	20	4	17	5	19
Practice	5	22	5	20	5	21	5	19
Government	1	4	1	4	1	4	1	4
Focus Areas^a^								
Family Physician Leadership for Health Equity	8	35	11	44	10	42	11	41
Diversity in Medicine	8	35	9	36	9	38	10	37
Health Equity Research in Primary Care	8	35	8	32	8	33	9	33
Public Policies for Health Equity	7	30	9	36	8	33	8	30

Note^: a^ Participants were assigned to more than one focus area

Abbreviations: MD, Doctor of Medicine; DO, Doctor of Osteopathic Medicine; PhD, Doctor of Philosophy; JD, Juris Doctor

### Health Equity priorities

Fifty priority statements were identified across the four focus areas, with vast textual support provided by the experts for qualitative data analysis. The findings for each focus area are presented below and the prioritized lists of statements are presented in Table 2.

**Table 2:** Statements Ranked by Focus Area

Rank	Strategy Statements	AR	SD	IQI
Focus Area 1: Leadership				5.7–7.4
What are the essential skills and characteristics family physicians need to become leaders for health equity?				
1	A deep and personal commitment to advancing health equity	3.0	3.7	
2	Knowledge of health inequities and their social, economic, and political drivers	4.3	3.0	
3	Knowledge of effective strategies to address social, economic, or political drivers of health inequities	4.5	3.4	
4	Advocating for public policy that aims to advance health equity	6.1	3.2	
5	Provide practice leadership to build a culture that values health equity	6.3	3.2	
6	Ability to engage disadvantaged communities by learning their needs and building their capacity and social capital	6.4	3.2	
7	Participation intersectoral partnerships or community-led teams to work collectively on common issues	6.7	2.6	
8	Speaking truth to power and holding institutions accountable to health equity	7.1	3.4	
9	Ability to use data to identify health inequities	7.2	2.5	
10	Screening patients for social determinants of health and referring them to appropriate community-based resources	8.0	2.3	
11	Physicians’ use of their status in society to advance health equity	8.6	3.6	
12	Provide practice leadership to maximize team-based care	9.8	2.5	
Focus Area 2: Policy				6.1–7.9
What public policies are the most important for improving health equity?				
1	Universal access to health insurance and high-quality, comprehensive health care	3.9	3.2	
2	Health in all Policies legislation or initiatives to ensure that policies that are traditionally considered outside of health (transportation, economics, etc.) are examined for their health implications before being voted on by legislative bodies	4.8	2.4	
3	Adequate funding for programs to supplement people’s incomes in times of need, such as welfare, unemployment insurance, and social security	6.0	3.5	
4	Policies that ensure equal employment opportunities for minorities	6.1	2.6	
5	Increasing the minimum wage to be greater than the poverty threshold	6.3	3.5	
6	Reinvestment in disadvantaged communities	6.5	3.6	
7	Policies that ensure environments are free from hazards in all communities	6.8	4.5	
8	Adequate funding for all public schools	6.9	4.4	
9	Policies to eliminate residential segregation, such as equitably dispersing low and moderate-income housing throughout metropolitan areas, as well as removing exclusionary zoning laws	7.0	3.0	
10	Adequate funding for center-based early childhood education for low-income families	7.9	3.8	
11	Value-based payment models to pay for performance and not fee-for-service	9.3	3.9	
12	Campaign finance reform to reduce the influence of the wealthy on political decisions	9.9	3.5	
13	Rescinded tax cuts to raise revenue to support public services	9.9	3.5	
Focus Area 3: Research				5.6–8.3
What are the most important areas of research to advance health equity?				
1	What multi-level interventions (clinical interventions and policy, systems, and environmental change) are effective at improving health equity and how can interventions at the individual and community levels be best coordinated?	3.7	2.5	
2	How do factors like policy, governance, and politics impact health equity and what can be done to change these factors to better support health for the vast majority of the population?	4.1	2.9	
3	How can effective interventions to advance health equity be translated into practice and scaled up for maximum reach?	4.4	2.8	
4	How can effective interventions for health equity be best disseminated to practitioners from a wide variety of disciplines to best promote collaboration across disciplines?	5.6	1.6	
5	How do racism and discrimination affect health and what strategies are effective for mitigating racism and discrimination and their effect on health?	5.9	2.6	
6	How can screening for social determinants of health in primary care best identify and address patient’s needs?	7.0	4.6	
7	What research methods are most appropriate for health equity research and in what context?	7.9	3.0	
8	What is health care’s role in advancing health equity considering that health inequities are caused primarily by factors like policy, governance, and politics?	8.0	3.7	
9	How can health equity be made more personally relevant to more people?	8.0	4.1	
10	What are the essential elements of effective intersectoral partnerships for health equity?	8.3	5.1	
11	How can unconscious biases be addressed by health care professionals to improve equity in health care quality?	8.9	3.3	
12	How can health equity data systems be improved to better measure things like within group heterogeneity, and health inequities in groups other than race, ethnicity, and social economic status?	9.4	3.3	
13	What payment model(s) promote(s) health equity?	9.9	3.8	
Focus Area 4: Diversity				5.7–7.7
What are the most important policies and practices to increase diversity in medicine?				
1	Provide financial support to students from minority or low socioeconomic backgrounds (tuition reimbursement, scholarships, grants, etc.) for college or medical school	3.3	2.8	
2	Commitment from undergraduate and medical school leadership for educational equity including formal goals and plans to intentionally recruit students and faculty from minority and lower socioeconomic backgrounds, and provide adequate resources (educational, financial, etc.) to support academic achievement	4.1	3.8	
3	Provide opportunities for students from minority or low socioeconomic backgrounds to prepare for standardized tests required for admission to college (ACT, SAT) or medical school (MCAT)	5.5	2.1	
4	Equitable primary and secondary school (grades K-12) funding to ensure schools that primarily serve students from minority or low socioeconomic backgrounds have sufficient financial resources to provide a high-quality education	5.8	4.5	
5	Address implicit bias among college and medical school admissions committees	6.1	3.0	
6	Provide assistance to students from minority or low socioeconomic backgrounds with navigating academia, such as assistance with completing college applications, writing personal statements, or developing CVs	6.8	2.6	
7	Provide tutoring support to students from minority or low socioeconomic backgrounds to maintain sufficient grades in college or medical school	6.9	4.1	
8	Develop programs to ensure low income students have their non-education-related financial needs met during college or medical school	7.0	3.6	
9	Identify and promote role modeling and mentoring by physicians who are minorities or from a low socioeconomic background	7.5	3.3	
10	Keep or implement affirmative action policies	8.1	2.0	
11	Strengthen the links between home and primary and secondary schools (grades K-12) to help disadvantaged parents help their children to learn	8.1	2.9	
12	Provide students from minority or low socioeconomic backgrounds targeted opportunities to build their extracurricular portfolio for applications to college or medical school	8.9	3.8	

Note: The results were calculated based on the third round of a three-round Delphi study

Abbreviations: *AR* Average Rank, *SD* Standard Deviation, *IQI* Interquartile Interval of Average Rank

#### Leadership: skills and characteristics physicians need to become leaders for Health Equity

Twelve skills and characteristics physicians need to be leaders for health equity were identified (Table 2). Commitment to health equity, knowledge of health inequities, and knowledge of effective strategies to address the drivers of health inequities were ranked the highest. Experts compared health equity leaders with medical leaders, saying that health equity leaders “have an implicit understanding that health equity is equivalent to health quality,” and that they “have a deliberate focus on cultural humility, self-reflection, [ …] and understanding of those who are different.” “Health equity leaders can effectively address the issues of race, racism, discrimination and the SDoH, all focused on improving life for the patient. Medical leaders focus on improving life for physicians and the institution they control.”

The next tier of statements included advocating for public policies to advance health equity, providing practice leadership to build a culture that values health equity, the ability to engage disadvantaged communities, and participation in intersectoral partnership or community-led teams. The experts discussed the importance of addressing both policy and patient issues and suggested that physician health equity leaders need a dual focus on both “clinical teams and community partnerships.” The experts also suggested that physician health equity leaders could bring “a different level of trust,” “personal stories,” and the ability to “debunk myths” that health inequities arise from “genetics […] group flaws, or behaviors.”

#### Policy: public policies for improving Health Equity

Thirteen public policy interventions to advance health equity were identified (Table 2). Universal access to health insurance and high-quality comprehensive health care were ranked the highest, and experts thought that the Affordable Care Act “took a step in the right direction.” However, experts cautioned that access to health insurance alone would not address the primary drivers of health inequities, saying “having universal health insurance is important but won’t do enough to address longstanding inequities based on [racism, education, environment, housing, pay, etc.].” Experts also cautioned that a medical approach may not be cost effective, saying “I’m concerned that current conversations about SDoH in health care [are too focused on social services]. We could end up with a very expensive social service sector without population health outcomes.”

Health in all policies was ranked a close second, and the remaining statements focused more on addressing the structural determinants of health inequities. The remaining statements focused on governmental decision making, providing resources to minority and low socioeconomic status populations, and equalizing opportunities throughout the social gradient. One expert suggested that the list was more important than the prioritization, saying “The 13 proposed strategies were all really important. It may make sense to have a few national policy priorities […] and offer the 13 strategies as examples of health equity policy, rather than trying to determine which are the most important.”

One expert captured the group’s opinion, saying:

“I think the most important thing is for the AAFP to engage its membership to be active voices for a platform that clearly articulates that health inequities are the result of historic and present institutionalized bias and discrimination that has shaped community conditions in ways that make people unhealthy. And, the solution to promoting health equity and wellbeing is supporting policies that improve community conditions, eliminate structural bias and discrimination, and promote equitable opportunities. Physicians should lend their voice to support community driven initiatives to achieve policy, systems, and environmental change strategies to promote health equity.”

#### Research: research to advance Health Equity

Thirteen health equity research topics were identified. Investigating which multi-level interventions are effective at improving health equity was ranked the highest. Other highly ranked statements focused on the effect of policy, governance, and politics; translating and disseminating health equity interventions into practice; and how to address the impacts of racism and discrimination on health. The experts thought that intervention research should be prioritized because health disparities are already well documented. Related to this, one expert said, “questions about which interventions work […] or do not work have not been answered. We have lots of evidence that describes problems […] but this does not lead directly to solutions.”

#### Diversity: policies and practices to increase diversity in medicine

Twelve policies and practices to increase diversity in medicine were identified (Table 2). The top ranked statements included providing financial support to students from minority or low-socioeconomic backgrounds, commitment from undergraduate and medical school leadership for educational equity, providing opportunities for students from minority or low-socioeconomic backgrounds to prepare for standardized tests, and equitable primary and secondary school funding. Many of the experts highlighted the need for structural solutions like improved school funding in urban and rural communities, saying “if children are empowered earlier, then [other interventions like Medical College Admission Test (MCAT) training] strike me as reactive.” However, some experts suggested there was a need for a mix of solutions, because structural interventions may take too much time to work. “It seems a bit overwhelming to think that we have to completely overhaul the K-12 education system before we can increase the diversity of medical students.” Connecting physicians with students from minority and low-socioeconomic backgrounds was commonly cited as a way to provide mentorship and role modeling. “If you don’t know doctors, it is unlikely you know how to become a doctor.”

## Discussion

While health inequities have been characterized in public health research for decades, research about effective interventions and best practices for prioritizing health equity interventions has lagged behind. Although more objective and quantifiable methods may be valuable for prioritization, this study using the Delphi technique provides one of the first attempts to rank the importance of issues across several health equity focus areas, providing information for decision makers in organizations aiming to advance health equity. Several important themes relevant to health equity planning emerged from this study, as described below.

### Leadership: stronger together

A large number of strategies were identified, with emphasis on that physician health equity leaders should work with their practice teams and community partners to advance health equity through clinical and community level interventions. Focus was placed on connecting clinical and public health interventions, building a practice culture that values health equity, and capacity to address SDoH. Addressing implicit bias in the practice and raising their cultural proficiency were highlighted, as was engaging in the broader community health agenda. Screening for SDoH and “community vital signs” were ranked relatively low even though these strategies have received attention in the medical literature [44, 45]. The importance of taking small, practical steps towards advancing health equity emerged as a key concept. The AAFP and other medical specialty societies can empower physicians to take action by providing education and learning opportunities, tools for practice and community engagement, and actionable information to help connect physicians with opportunities for advancing health equity.

### Policy: advocacy

The importance of advocacy for health equity emerged as a prominent concern, as it was raised across all focus areas and ranked highly. The AAFP, like most medical specialty societies advocate on behalf of their members and have strong connections with federal and state policy makers. The AAFP and other medical specialty societies have an important opportunity to address the SDoH by developing a health equity policy agenda using a health in all policies approach [25]. The impact of policies from outside of the medical sector on health are widely known and the data from this study reiterate this concern [43]. While this study assumed that there would be a natural prioritization among the strategies proposed, some of the experts resisted this idea, suggesting that the health equity policy agenda as a whole is more important. Using the policy interventions and priorities identified in this study as a guide, the AAFP and other medical specialty societies could develop a health equity policy agenda that prepares them to take decisive action as policy windows emerge at the national, state, and local levels [25].

### Research: focus on solutions

A variety of research needs were identified, including those related to the nature and focus of interventions, dissemination and translation, as well as research methods and data systems. These findings are well aligned with calls to action for health equity research published previously [10, 46–48]. Experts in this study indicated that focus for health equity research should shift from identifying and measuring health disparities, to testing and translating interventions into practice. More focus and new research designs are needed to address the root structural causes of health inequities that have been resistant to change [49]. Practice-based research, including systems science and qualitative research may be more effective for studying complex issues like creating effective partnerships, or leading policy and systems change that are essential for advancing health equity [10, 50, 51]. The AAFP, like many medical specialty societies, has relationships with practice-based research networks, and are primed to help close these gaps [52]. Again, funding is needed to support practice-based research for health equity and the Federal Government, foundations, and other funders should consider creating funding portfolios to support more of this work [48].

### Diversity: level the playing field

The field of medicine overall lacks diversity [53]. Increasing diversity is critical to address health equity for a number of reasons, including that clinicians from minority and low-socioeconomic backgrounds are more likely to serve disadvantaged communities [26, 53, 54]. The findings from this study suggest that both a mix of structural interventions that remove systematic barriers as well as proximal interventions which can have a more immediate impact and produce the momentum that is needed. Medical specialty societies, like the AAFP, should advocate for increased diversity in medicine by placing pressure on the Federal Government and other funders to provide financial support to minority and low-income students and compelling medical schools to develop strategic plans for diversifying the physicians that they graduate. As part of community engagement, physician health equity leaders should connect with minority and low-socioeconomic communities to help raise awareness of and preparedness for opportunities among students for entering medical school.

### Limitations

The Delphi technique and analysis used herein posed a few limitations [55]. First, developing a list of independent strategy statements from the participants’ input and literature review was challenging because health equity issues are complex and interrelated. However, comprehensive lists of fairly distinct strategies were identified for each focus area. Second, Delphi study guidelines suggest that more than 10 experts be included, however two of the focus areas did not meet the 10-participant threshold (research and policy). However, twenty-seven experts were included in the overall study and it was possible to triangulate common themes across focus areas to strengthen the findings. In addition, the qualifications of the participants were strong and included a rich mixture of highly educated and experienced individuals with proven leadership from academia and practice as well as from medicine and public health. Finally, ordinal ranks did not produce dispersion and forced ranking was used to identify priority strategies instead. While this is not necessarily a limitation, it differs from the majority of studies using the Delphi technique and the strategy statements were prioritized rather than reaching consensus.

## Conclusion: implications for health equity

Health inequities are differences in health outcomes that are systematic, avoidable and unjust [1]. To advance health equity, society must work to address their underlying drivers, such as systemic discrimination and racism that manifests in institutional procedures and public policies. Medical specialty societies, like the AAFP, are well positioned to take leadership role in advancing health equity since they have influence with clinicians, health care organizations, and policy makers. As a burgeoning leader of health equity, the AAFP has established a four-pronged strategy to leverage its strengths to advance health equity. This includes: equipping family physicians with information and resources to address the SDoH that drive health inequities; advocating for public policies that can improve health equity; promoting and shaping the research agenda for health equity; and increasing diversity in medicine. The findings from this study have informed the AAFP’s health equity strategy, helping to better align resources with opportunities. While additional research is needed to provide a tangible measure of the impact or likelihood of success for a broad set of health equity strategies, the information provided from this study should be able to help inform other organization’s health equity strategies.

## Additional file

Additional file 1: Strategy statements identified in the first round of the Delphi study. (DOCX 26 kb)

## Data Availability

The datasets generated and analyzed in this study are not publicly available to ensure participant confidentiality.
