# Supplementary material for: Informing the American Academy of family Physician’s Health Equity strategy – an environmental scan using the Delphi technique
Source: Int J Equity Health. 2019 Jun 21;18:97. doi: 10.1186/s12939-019-1007-1 (PMC6588858; doi:10.1186/s12939-019-1007-1)
Supplement: Supplementary file 1 — Strategy statements identified in the first round of the Delphi study. (DOCX 26 kb) [file 12939_2019_1007_MOESM1_ESM.docx]

**AMERICAN ACADEMY OF FAMILY PHYSICIANS**

**HEALTH EQUITY DELPHI STUDY**

**ROUND ONE STRATEGY STATEMENTS**

**Developing Family Physician Health Equity Leaders**

| ***Strategy Statements*** |
| --- |
| 1. Knowledge of health inequities and their drivers. |
| 1. Ability to use data to identify health inequities. |
| 1. Ability to use stories to illustrate the effect of social determinants on health. |
| 1. Knowledge of effective strategies to address social, economic, or political drivers of health inequities. |
| 1. A deep and personal commitment to advancing health equity. |
| 1. A personal commitment to self-evaluation to redressing the power imbalances in the patient and physician dynamic. |
| 1. The trained ability to discern how symptoms, health behaviors, and diseases also represent the downstream implication of a number of upstream decisions about such matters as politics, governance and laws, the built environment and infrastructure, education, zoning, health care access and delivery, etc. |
| 1. Leadership of intersectoral partnerships to work collectively on common issues. |
| 1. Ability to engage disadvantaged communities by learning their needs and building their capacity and social capital. |
| 1. Speaking truth to power and holding institutions accountable to health equity. |
| 1. Physicians’ use of their status in society to advance health equity. |
| 1. Prolific communication about health equity. |
| 1. Adaptation of communication to meet patient’s needs (ask me three, teach back, etc.). |
| 1. Provide practice leadership to build a culture that values health equity. |
| 1. Provide practice leadership to maximize team-based care. |
| 1. Screening patients for social determinants of health and referring them to appropriate community-based resources. |
| 1. Advocating for public policy that aims to advance health equity. |

**Increasing Diversity in Medicine**

| **Strategy Statements** |
| --- |
| 1. Commitment from primary and secondary school (grades K-12) leadership for educational equity including formal goals and plans to advance educational equity for minorities and individuals from a low socioeconomic background. |
| 1. Commitment from undergraduate and medical school leadership for educational equity including formal goals and plans to intentionally recruit students and faculty from minority and lower socioeconomic backgrounds, and provide adequate resources (educational, financial, etc.) to support academic achievement. |
| 1. Ensure that funding levels are adequate for all primary and secondary schools (grades K-12) and allocate funds based on student need. |
| 1. Equitable primary and secondary school (grades K-12) funding to ensure that schools that primarily serve students from minority or low socioeconomic backgrounds have sufficient financial resources to provide a high-quality education. |
| 1. Ensure equitable opportunities for students from minority or low socioeconomic backgrounds to participate in advanced placement courses in primary and secondary education (grades K-12). |
| 1. Manage school choice in primary and secondary schools (grades K-12) so to contain the risks to equity. |
| 1. Strengthen the links between primary and secondary schools (grades K-12) and home to help disadvantaged parents help their children to learn. |
| 1. Provide opportunities for students from minority or low socioeconomic backgrounds to prepare for standardized tests required for admission to college (ACT, SAT) or medical school (MCAT). |
| 1. Keep or implement affirmative action policies. |
| 1. Identify and promote role modeling and mentoring by physicians that are minorities or from a low socioeconomic background. |
| 1. Provide opportunities for students from minority or low socioeconomic backgrounds to shadow physicians to gain exposure to medicine as a profession. |
| 1. Provide students from minority or low socioeconomic backgrounds assistance navigating academia, such as assistance with completing college applications, writing personal statements, or developing CVs. |
| 1. Provide students from minority or low socioeconomic backgrounds tutoring support to maintain sufficient grades in college or medical school. |
| 1. Improve science, technology, engineering, and math programs in primary and secondary schools (grades K-12) that primarily serve students from minority or low socioeconomic backgrounds. |
| 1. Provide students from minority or low socioeconomic backgrounds financial support (tuition reimbursement, scholarships, grants, etc.) for college or medical school. |
| 1. Provide students from minority or low socioeconomic backgrounds targeted opportunities to build their extracurricular portfolio for applications to college or medical school. |
| 1. Address implicit bias among college and medical school admissions committees. |
| 1. Develop programs to ensure low income students have their non-education-related financial needs met during college or medical school. |

**Improving Public Policy To Support Health Equity**

| **Strategy Statements** |
| --- |
| 1. Improved access to health insurance and high-quality, comprehensive health care. |
| 1. Value-based payment models to pay for performance and not fee-for-service. |
| 1. Adequate funding for governmental public health organizations in all communities. |
| 1. Adequate funding to support home visiting programs for pregnant women and families with young children. |
| 1. Adequate funding to support data collection methods that are standardized and that allow for health inequities to be identified at small geographic levels (counties, cities, zip-codes, etc.). |
| 1. Adequate funding to support nutrition assistance programs, such as WIC and SNAP. |
| 1. Policies that ensure environments are free from hazards in all communities. |
| 1. Policies to reduce morbidity and mortality from firearms. |
| 1. Adequate funding for programs to supplement people’s incomes in times of need, such as welfare, unemployment insurance, and social security. |
| 1. Policies that ensure people are permitted to be absent from their work in time of need, such as the Family Medical Leave Act, as well as paid sick leave and paid parental leave. |
| 1. Health in all Policies legislation or initiatives to ensure that policies that are traditionally considered outside of health (transportation, economics, etc.) are examined for their health implications before being voted on by legislative bodies. |
| 1. Adequate funding to support intersectoral initiatives to develop and implement plans to improve the public’s health (i.e., community health assessment, community health improvement planning, community health needs assessment, state innovation models, etc.). |
| 1. Criminal justice reforms, including race-neutral sentencing, adequately funded re-entry programs and public defenders, and eliminating questions about a criminal record for employment and welfare programs. |
| 1. Policies to eliminate residential segregation, such as equitably dispersing low and moderate-income housing throughout metropolitan areas, and removing exclusionary zoning laws. |
| 1. Policies and funding to ensure civil rights laws are adequately enforced. |
| 1. Policies to ensure that disadvantaged communities can participate in governmental decision making, such as providing information and seeking feedback. |
| 1. Increasing the minimum wage to be greater than the poverty threshold. |
| 1. Policies that improve employment opportunities for all people. |
| 1. Policies that ensure equal employment opportunities for minorities. |
| 1. Rental assistance programs to allow low-income families better housing options. |
| 1. Earned income tax credit and other progressive taxation policies. |
| 1. Occupational safety laws and funding. |
| 1. Adequate funding for all public schools. |
| 1. Full-day kindergarten programs. |
| 1. High school completion programs. |
| 1. Adequate funding for center-based early childhood education for low-income families. |
| 1. Adequate funding for out-of-school-time academic programs with an educational component. |
| 1. School-based health centers. |
| 1. Policies that support a healthy built environment, including walkable streets, adequate public transportation, parks and recreational opportunities, etc. |
| 1. Campaign finance reform to reduce the influence of the wealthy on political decisions. |
| 1. Comprehensive immigration reform so that immigrants and their families can access employment, governmental programs, etc. |
| 1. Adequate funding for translation language services. |
| 1. Rescinded tax cuts to raise revenue to support public services. |
| 1. Reinvestment in disadvantaged communities. |

**Health Equity Research**

| **Strategy Statement** |
| --- |
| 1. How has globalization impacted health equity and what are the implications for population health at the local, state, national, and global levels? |
| 1. How can health equity data systems be improved to better measure things like within group heterogeneity, and health inequities in groups other than race, ethnicity, and social economic status? |
| 1. How can health equity be made more personally relevant to more people? |
| 1. How do factors like policy, governance, and politics impact health equity and what can be done to change these factors to better support health for the vast majority of the population? |
| 1. What is health care’s role in advancing health equity considering that health inequities are caused primarily by factors like policy, governance, and politics? |
| 1. What multi-level interventions are effective at improving health equity and how can interventions at the individual and community levels be best coordinated? |
| 1. How do racism and discrimination affect health and what strategies are effective for mitigating racism and discrimination and their effect on health? |
| 1. What research methods are most appropriate for health equity research and in what context? |
| 1. How can effective interventions to advance health equity be translated into practice and scaled up for maximum reach? |
| 1. What health care funding, delivery, and management models best support comprehensiveness of services and equity in access? |
| 1. What technological solutions can help advance health equity? |
| 1. What would an infrastructure for health equity be comprised of and how could this be financed and developed? |
| 1. How can effective interventions for health equity be best disseminated to practitioners from a wide variety of disciplines to best promote collaboration across disciplines? |
| 1. How can unconscious biases be addressed by health care professionals to improve equity in health care quality? |
| 1. What are the essential elements of effective intersectoral partnerships for health equity? |
| 1. How can screening for social determinants of health in primary care best identify and address patient’s needs? |
| 1. What payment model(s) promote health equity? |
